# A Novel Ultrasensitive Single‐Molecule Counting Technology for Quantitation of Neurological Biomarkers in Blood

**DOI:** 10.1002/alz.095782

**Published:** 2025-01-09

**Authors:** Renee Tobias, Peter Wagner, Johanna Sandlund, Katsumi Aoyagi, Kazushige Moriyama, Frank Zaugg, Gipshu Dave, Daigo Inaoka, Hayato Kimura, Ko Kobayashi, Valerie Brachet

**Affiliations:** ^1^ Fluxus, Inc, Sunnyvale, CA USA; ^2^ Fujirebio, Inc, Tokyo, Tokyo Japan; ^3^ Fujirebio Inc., Tokyo Japan

## Abstract

**Background:**

There is a need for sensitive, robust, and scalable analytical methods for accurate, early detection of disease using less invasive sampling methods. Small volumes and low marker concentrations present challenges to achieving sufficient sensitivity and accuracy with traditional immunoassay methods ‐ particularly when measuring markers for Alzheimer’s (AD) and other neurological diseases in blood, where levels are typically much lower than in CSF.

Here we introduce a novel optofluidic single‐molecule counting platform for ultrasensitive detection of low‐abundance biomarkers in plasma samples. Analytical validation data is presented for three Alzheimer’s Disease plasma biomarker assays developed on a fully automated benchtop analyzer with integrated ultrasensitive (US) detector.

**Method:**

Fluorescence‐based immunoassays for pTau181, Aβ1‐40, and Aβ1‐42 were developed using high performance monoclonal antibodies and reagents (Fujirebio Inc, Japan). Capture antibodies were immobilized on magnetic particles, and detection antibodies conjugated to a proprietary fluorescent reporter construct. Multiple incubation and wash steps were followed by dissociation of immune complexes and separation of reporter‐Ab from magnetic particles. Released reporter‐Ab molecules were injected into the US detector device for single molecule counting.

**Result:**

We performed analytical performance evaluation of each of the three assays, assessing sensitivity (LOD/LLOQ), linearity, parallelism, precision, and repeatability with clinical samples. Assay LLOQs of endogenous analyte spiked in plasma matrix were estimated at 0.2 pg/mL, 0.5 pg/mL, 0.5 pg/mL respectively for pTau181, Aβ1‐40, and Aβ1‐42 markers. Comparison to Lumipulse G1200 Plasma Tests for pTau181, Aβ1‐40, and Aβ1‐42 (Fujirebio Diagnostics, Inc.) yielded correlation coefficients of >0.998 through a working range of >4 logs.

**Conclusion:**

We have developed a new single‐molecule counting platform able to detect biomarkers at sub‐pg/ml levels, with the potential to provide sensitive and accurate diagnostic testing of low abundance neurological markers in blood with improved performance and reproducibility over currently available methods. Such technologies can create new clinical value through higher detection sensitivity and accuracy in areas of need such as Alzheimer’s and other neurological diseases, to support clinical research and translation to clinical practice.

